# Immune Profiling of Patients with Systemic Sclerosis through Targeted Proteomic Analysis

**DOI:** 10.3390/ijms242417601

**Published:** 2023-12-18

**Authors:** Iulia Szabo, Medeea Badii, Ildikó O. Gaál, Robert Szabo, Claudia Sîrbe, Oana Humiță, Leo A. B. Joosten, Tania O. Crișan, Simona Rednic

**Affiliations:** 1 Department of Rheumatology, “Iuliu Hațieganu” University of Medicine and Pharmacy, 400012 Cluj-Napoca, Romania; rotaru.iulia@umfcluj.ro (I.S.);; 2 Department of Rheumatology, County Emergency Hospital, 400347 Cluj-Napoca, Romania; 3 Department of Medical Genetics, “Iuliu Hațieganu” University of Medicine and Pharmacy, 400012 Cluj-Napoca, Romania; 4 Department of Internal Medicine, Radboud Institute for Molecular Life Sciences (RIMLS), Radboud University Medical Center, 6525 GA Nijmegen, The Netherlands; 5 2nd Anesthesia Department, “Iuliu Hațieganu” University of Medicine and Pharmacy, 400012 Cluj-Napoca, Romania; 6 Department of Anesthesia and Intensive Care, County Emergency Hospital, 400347 Cluj-Napoca, Romania; 7 2nd Pediatric Discipline, Department of Mother and Child, “Iuliu Hațieganu” University of Medicine and Pharmacy, 400012 Cluj-Napoca, Romania; 8 2nd Pediatric Clinic, Center of Expertise in Pediatric Liver Rare Disorders, Emergency Clinical Hospital for Children, 400177 Cluj-Napoca, Romania

**Keywords:** systemic sclerosis, proteomics, inflammatory endotype, chemokines, TNF

## Abstract

High-throughput proteomic analysis could offer new insights into the pathogenesis of systemic sclerosis (SSc) and reveal non-invasive biomarkers for diagnosis and severity. This study aimed to assess the protein signature of patients with SSc compared to that of healthy volunteers, decipher various disease endotypes using circulating proteins, and determine the diagnostic performance of significantly expressed plasma analytes. We performed targeted proteomic profiling in a cohort of fifteen patients with SSc and eighteen controls using the Olink^®^ (Olink Bioscience, Uppsala, Sweden)Target 96 Inflammation Panels. Seventeen upregulated proteins involved in angiogenesis, innate immunity, and co-stimulatory pathways discriminated between patients with SSc and healthy controls (HCs) and further classified them into two clusters, a low-inflammatory and a high-inflammatory endotype. Younger age, shorter disease duration, and lack of reflux esophagitis characterized patients in the low-inflammatory endotype. TNF, CXCL9, TNFRSF9, and CXCL10 positively correlated with disease progression, while the four-protein panel comprising TNF, CXCL9, CXCL10, and CX3CL1 showed high diagnostic performance. Collectively, this study identified a distinct inflammatory signature in patients with SSc that reflects a persistent T helper type 1 (Th 1) immune response irrespective of disease duration, while the multi-protein panel might improve early diagnosis in SSc.

## 1. Introduction

Systemic sclerosis (SSc) is a chronic autoimmune disease characterized by a heterogeneous clinical phenotype and a high risk of mortality mainly determined by the occurrence of major internal organ complications, such as interstitial lung disease (ILD) and pulmonary arterial hypertension (PAH). Its complex pathogenesis, marked by the interplay among immune dysregulation, microvascular alterations, and fibrosis, is not completely unraveled. A proteomic analysis can not only offer a better understanding of the mechanistic pathways, but also address the unmet need for non-invasive biomarkers for SSc diagnosis, assessments of disease severity, prognosis, and the response to treatment. In a systematic review published in 2021 [1], 539 candidate proteomic biomarkers were identified from various biological samples in patients with SSc using mass spectrometry with only 13 analytes (S100A8/A9, UCHL1, PPID, DDX55, COX6B1, APCS, 14-3-3 epsilon, S100A6, collagen-1, vimentin, KRT17, SDF4, MZB1) being validated in independent cohorts. In another report, SOMAscan aptamer technology was used for proteomic profiling, and 8 of the 181 differentially expressed circulating proteins were validated in 14 diffuse cutaneous SSc (dcSSc) patients and correlated with the modified Rodnan Skin Score (mRSS) [2]. In a more recent study, 10 of the 286 most significantly expressed serum proteins were strongly associated with disease progression from preclinical to definite SSc. In the validation cohort (50 preclinical SSc patients), endostatin, basic fibroblast growth factor (bFGF), and platelet-activating factor acetylhydrolase-β subunit (PAF-AHβ), proteins related to angiogenesis and fibrosis processes, were particularly associated with the risk of developing definite SSc [3]. A secretome analysis of circulating exosomes from four patients with primary Raynaud’s phenomenon and three patients with Raynaud’s phenomenon at risk of progressing to SSc identified 22 differentially elevated or reduced proteins involved in inflammatory processes and associated with vasculopathy [4]. Chemerin was confirmed to be significantly elevated in the serum of 15 systemic sclerosis-associated pulmonary arterial hypertension (SSc-PAH) patients compared to levels in 16 SSc patients without PAH in both the discovery and validation cohorts and was highly correlated with pulmonary vascular resistance (PVR) values, and hence, this protein was proposed as a biomarker of hemodynamic severity in SSc-PAH [5]. The previously published data are highly heterogeneous, and an important caveat is the variation in sample sizes, biological specimens, and technologies used. Another limitation in interpreting the clinical utility of proteomics is the lack of data concerning the concordance of between-sample similarities. The multi-omic comparative analysis of SSc serum, peripheral blood cells (PBCs), and skin performed by Farutin et al. [6] addressed this issue and proved that the serum proteome is correlated more closely with the skin transcriptome than corresponding transcripts in PBCs, thus revealing the potential role of serum proteins to be used as surrogate markers of disease severity at the organ level.

Nevertheless, serum and plasma samples remain an attractive and non-invasive source for assessing proteomic data, and antibody-based methods, such as the OLINK platforms, offer higher target specificity, thereby enhancing the ability of uncovering more reliable phenotypic associations [7]. This study aimed to evaluate the plasma protein profiles of patients with SSc compared to those of HCs, identify disease endotypes based on unique proteomic signatures, and analyze the performance of circulating proteins as diagnostic biomarkers.

## 2. Results

### 2.1. Demographic and Clinical Characteristics of the Study Participants

The baseline clinical and laboratory features of patients with SSc are depicted in Table 1. Two SSc samples did not pass Olink^®^’s integrated Quality Control (QC) checks and were removed from subsequent analysis. One patient was diagnosed with an overlap with myositis and was excluded from the study. All enrolled SSc subjects (*n* = 15) and healthy volunteers (*n* = 18) were females with no age differences between patients, 53 (48, 60) years, and controls, 49.5 (42, 56) years (*p* = 0.1514). All patients received immunomodulatory therapy, including methotrexate, azathioprine, hydroxychloroquine, leflunomide, mycophenolate mofetil, cyclophosphamide, or tocilizumab.

A subgroup of five patients exhibited a proteomic profile comparable to that of HCs. In this subset of study participants, the median and inter-quartile range (IQR) age was 42 (37, 51.5) years. According to the extent of skin involvement, three (60%) had dcSSc. The median disease duration was 2 (0.5, 4.5) years, with 3 (60%) of patients having early (≤3 years) disease. ILD was present on high-resolution computed tomography (HRCT) in two (40%) patients with SSc. A previous history of digital ulcers was registered in two (40%) patients. Cardiac involvement, PAH, and esophagitis did not occur in this cohort. Antinuclear antibodies (ANA) were positive in all patients, with three (60%) having anti-Scl-70 antibodies and two (40%) exhibiting anti-centromere antibodies.

### 2.2. Patients with SSc Demonstrate a Distinct Proteomic Profile

**Figure 1:**
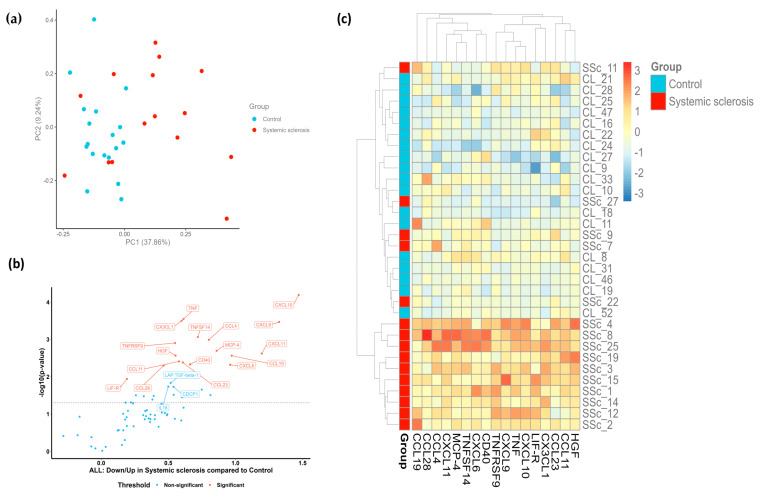
Targeted proteomic profiling of patients with SSc compared to healthy volunteers. (**a**) PCA plot of patients with SSc (red dots) and HCs (blue dots). Each dot represents a sample. The percentage of variation accounted for each principal component is shown in brackets along the *x*- and *y*-axis labels. (**b**) Volcano plot representation of differentially expressed proteins in patients with SSc compared to levels in HCs. Each dot represents a protein. A Welch two-sample *t*-test at a confidence level of 0.95 was performed for every protein. (**c**) Heatmap of significantly upregulated proteins in SSc samples. Columns represent individual proteins, while rows represent individual samples. Row and column dendrograms show the distance/similarity between the variables. The relative value for each protein is shown based on the color intensity of the Z score, ranging from low (blue) to high (red).

Proteins with Normalized Protein eXpression (NPX) values below the limit of detection (LOD) in more than 50% of the samples were excluded from the comparative analysis. Based on the quantitative features of the remaining 71 plasma proteins, the principal component analysis (PCA) plot of the 15 SSc samples and 18 control samples revealed that patients with SSc display a distinct proteomic signature compared to their matched controls (Figure 1a). A further assessment of differentially expressed proteins in patients with SSc versus HCs resulted in the identification of 17 plasma biomarkers with a false discovery rate (FDR)-adjusted *p*-value < 0.05. These 17 inflammation-related proteins were all upregulated in the SSc cohort compared to levels in the control group (Figure 1b). Following the hierarchical clustering of differentially abundant proteins, a prominent separation between groups was observed, with the majority of the SSc samples clustering together in the lower section of the heatmap (Figure 1c). Figure 1c also clearly depicts the subset of five individuals with SSc described above that showed protein profiles that closely resembled those of the control group. The heterogeneity within the SSc group might suggest the potential presence of different molecular subtypes or disease endotypes.

### 2.3. Portrayal of Inflammatory Endotypes Based on the Identified Protein Signature

**Figure 2:**
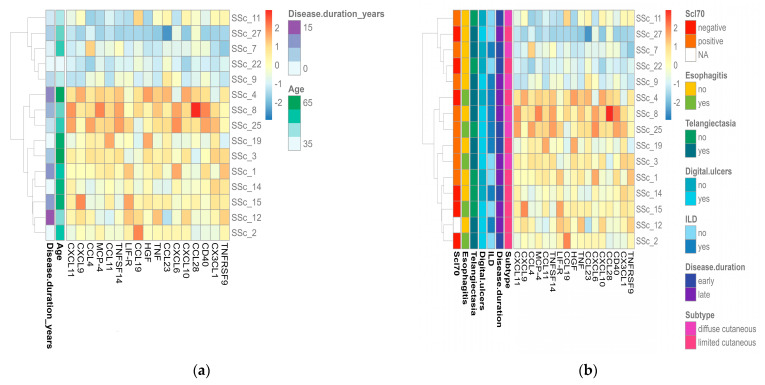
Visual representation of SSc endotypes in relation to protein abundances and clinical characteristics. (**a**) Heatmap of significantly upregulated inflammatory proteins in patients with SSc according to age and disease duration. (**b**) Heatmap of significantly upregulated inflammatory proteins in patients with SSc according to disease subtype, internal organ involvement, and laboratory features. Columns represent individual proteins, while rows represent individual samples. Row and column dendrograms show the distance/similarity between the variables. The relative value for each protein is shown based on the color intensity of the Z-score, ranging from low (blue) to high (red).

We performed unsupervised clustering of patients with SSc based on the 17 significantly expressed proteins and identified two molecular endotypes (Figure 2). Cluster 1 (*n* = 5) comprised patients with low protein abundances, as opposed to cluster 2 (*n* = 10), which was characterized by higher protein abundances. Interestingly, the protein expression profiles of patients included in the low-inflammatory endotype resembled those of controls (Figure 1c). Compared to the rest of the SSc cohort, the distinguishing features of the patients from cluster 1 were a younger age and shorter disease duration (Figure 2a), as well as the lack of endoscopic findings suggestive of esophagitis (Figure 2b). When analyzing disease duration from the perspective of early (≤3 years) versus late (>3 years) SSc, no difference could be observed between the two clusters. No other relevant differences in clinical manifestations or laboratory parameters could be observed between the low- and high-inflammatory endotypes.

### 2.4. Inflammatory Proteins Are Significantly Upregulated in Patients with a Longer Disease Duration

**Figure 3:**
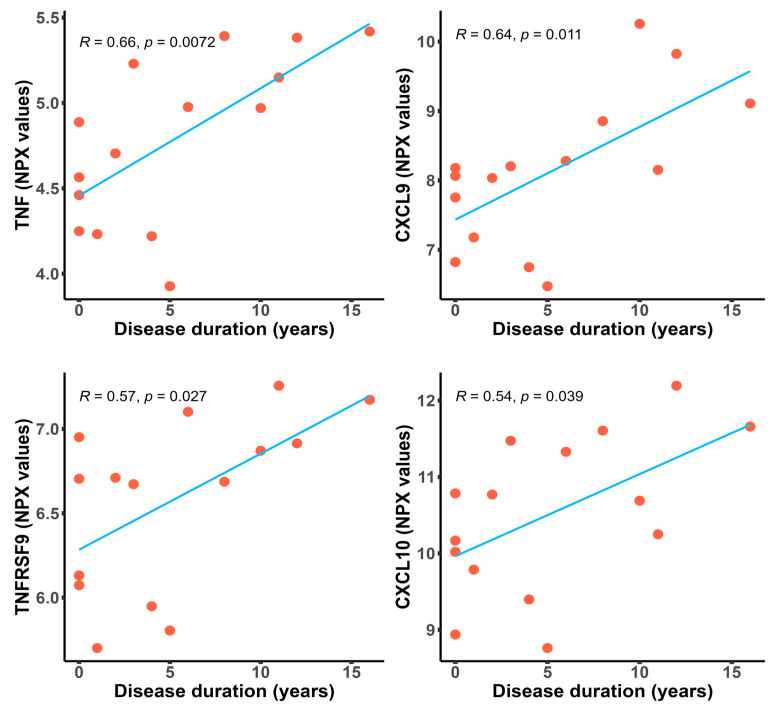
Scatter plots depicting positive correlations between the NPX values of significantly expressed proteins and disease duration in patients with SSc, with the Pearson’s correlation coefficient. The fitted linear regression line of each scatter plot (blue) is depicted along with the R and *p*-values.

In SSc, research is focused mainly on early disease, as most of the organ involvement and skin fibrosis develop in the first two to five years from the first non-Raynaud symptom [8,9,10]. Lately, emerging evidence suggests that severe complications can also occur at a later point in time [11,12,13,14]. We assessed the relationship between differentially expressed proteins in patients with SSc and disease duration (Table 2). As shown in Figure 3, TNF (*R* = 0.66, *p* = 0.0072), CXCL9 (*R* = 0.64, *p* = 0.011), TNFRSF9 (*R* = 0.57, *p* = 0.027), and CXCL10 (*R* = 0.54, *p* = 0.039) were positively correlated with disease progression. Surprisingly, further comparative proteomic profiling of patients with early disease (≤3 years) versus late disease (>3 years) did not identify any significant differences in the expression levels of proteins.

### 2.5. Performance of Differentially Expressed Proteins for the Diagnosis of SSc

**Figure 4:**
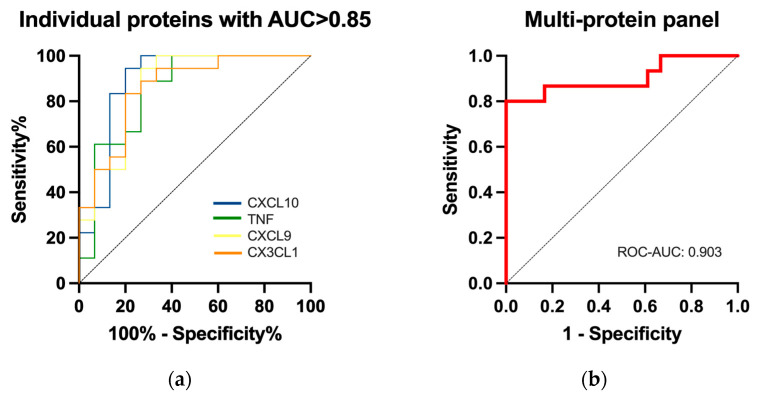
Predictive accuracy of circulating biomarkers to discriminate between patients with SSc and HCs. (**a**) Logistic ROC curves of individual proteins. (**b**) Logistic ROC curve of the multi-protein (CXCL10, TNF, CXCL9, CX3CL1) panel. ROC, receiver operating characteristic; ROC-AUC, the area under the ROC curve.

The discriminatory ability of the seventeen circulating inflammatory biomarkers was assessed through receiver operating characteristic (ROC) curve analysis. High diagnostic accuracy, with an area under the curve (AUC) > 0.85, was registered for the following four analytes: CXCL10 (AUC = 0.888, *p* = 0.0001, sensitivity = 94.4%, specificity = 80.0%), TNF (AUC = 0.851, *p* = 0.0006, sensitivity = 88.8%, specificity = 73.3%), CXCL9 (AUC = 0.870, *p* = 0.0003, sensitivity = 83.3%, specificity = 80.0%), and CX3CL1 (AUC = 0.859, *p* = 0.0005, sensitivity = 83.3%, specificity = 80.0%) (Figure 4a). The best diagnostic performance was, however, obtained using the four-protein panel (AUC = 0.903, *p* < 0. 0001, sensitivity = 86.7%, specificity = 83.3%) (Figure 4b).

## 3. Discussion

Immune alterations are a prominent culprit in SSc pathogenesis. Innate immune activation, the loss of self-tolerance, the production of highly specific and mutually exclusive autoantibodies, and evidence of an “interferon signature” are key processes shown to modulate both vasculopathy and fibrosis [15]. Recently employed single-cell RNA sequencing techniques and various “omics” approaches confirm the complexity and heterogeneity of the disease [16,17], thus supporting more research in the field of inflammation and immune dysregulation.

In this study, we applied the antibody-based Olink^®^ assay to identify distinct inflammatory profiles in SSc and investigate the diagnostic and classification performance of circulating proteins. Targeted proteomic profiling of the 92 inflammatory analytes included in the panel revealed that patients with SSc have a distinct protein signature compared to that of healthy volunteers. The protein abundances of the seventeen differentially expressed plasma analytes were further used to categorize patients with SSc into a low-inflammatory and a high-inflammatory endotype. Four circulating proteins (TNF, CXCL9, TNFRSF9, and CXCL10) were positively correlated with disease duration, suggesting a persistent Th 1 response as the disease progresses. Moreover, TNF, CXCL9, CXCL10, and CX3CL1 showed diagnostic potential with the best predictive accuracy being obtained when using the four-protein panel.

As mentioned earlier, a discrete plasma protein profile was identified in our SSc cohort consisting of 17 upregulated proteins (Figure 1a–c). Among those, we observed a prominent chemokine signaling signature, as most upregulated proteins belong to the CXC and CC chemokine families. These molecules display a strong chemotactic activity for different leucocyte subsets to the inflammation site [18]. CXC chemokines containing the ELR motif (Glu-Leu-Arg), such as CXCL6, have proangiogenic properties, while interferon-gamma (IFN-γ) inducible chemokines lacking the ELR motif are potent inhibitors of angiogenesis (CXCL9, CXCL10, CXCL11) [19]. Members of the CC chemokine family (CCL19, MCP-4/CCL13, CCL11), as well as CX3CL1, the only member of the CX3C subfamily, are involved in immune cell recruitment and extravasation, thereby promoting vascular injury [6,20,21,22]. Notably, there was limited data availability in the existing literature for several of the identified proteins (CCL11/eotaxin-1, CCL28, LIF-R, TNFRSF9) and their association with a potential pathogenetic role in SSc. Accordingly, there is no supporting evidence of the involvement of CCL28 in the development or progression of SSc. However, Sivakumar et al. performed a comparative analysis of the lung transcriptome and plasma proteome in patients with idiopathic pulmonary fibrosis (IPF) and demonstrated that CCR10, the receptor for CCL28, was upregulated in lung tissues and correlated with elevated plasma levels of CCL28 [23]. Previous studies have demonstrated that CCR10+ epithelial cells are robustly expressed in IPF lungs. In vitro experiments showed that these cells can induce IPF lung fibroblast invasion and collagen 1 secretion while promoting lung remodeling in humanized mice [24]. Taken together, these findings highlight a role for the signaling pair CCL28/CCR10 in promoting pulmonary fibrosis and enables us to hypothesize that the CCL28/CCR10 axis could also drive fibrosis in SSc.

The tumor necrosis factor (TNF) superfamily (TNFSF) and TNF receptor superfamily (TNFRSF) were also well represented in our patient population (TNF, TNFSF14, TNFRSF9/CD137, CD40/TNFRSF5). TNF is primarily produced by monocytes and macrophages, innate immune cells able to acquire adaptive characteristics after exposure to certain exogenous or endogenous insults, resulting in enhanced responsiveness to subsequent non-specific stimuli. This concept is referred to as innate immune memory or trained immunity [25,26]. Trained immunity is characterized by a metabolic shift towards increased aerobic glycolysis, oxidative phosphorylation (OXPHOS), tricarboxylic acid (TCA) cycle activity, glutaminolysis, and fatty acid metabolism with the synthesis of certain metabolites capable of modulating the DNA methylation status of genes involved in the innate immune responses [25,26]. Thus, metabolic, and epigenetic rewiring enable a faster, stronger, and long-lasting response upon a second challenge. Besides its beneficial effects against infections, endogenous stimuli could induce aberrant immune responses leading to tissue damage and chronic inflammation [25,26]. In this regard, a growing body of evidence has suggested that trained immunity-like processes could contribute to the immune dysregulation seen in patients with SSc [27,28]. Another interesting observation is the potential involvement of TNFSF14/lymphocyte T-related inducible ligand that competes for glycoprotein D binding to herpes virus entry mediator on T cells (LIGHT) in innate defense mechanisms [29]. Gindzienska-Sieskiewicz and colleagues recently showed that LIGHT and its receptors, herpes virus entry mediator (HVEM) and lymphotoxin β-related receptor (LTβR), are highly expressed in SSc skin biopsies, while serum concentrations of LIGHT were associated with the presence of digital ulcers [30]. In keeping with the role of co-stimulatory pathways in shaping the immunological responses in SSc, the CD137 (TNFRSF9) receptor is highly expressed on circulating monocytes, and upon its stimulation, a shift towards a metabolically more active phenotype with the upregulation of both glycolysis and OXPHOS occurs, as well as an increase in monocyte proinflammatory activity and phagocytosis [31]. Similarly, the CD40 axis not only promotes fibroblast activation but is also involved in innate memory responses [32,33]. Overall, further exploration of the receptor–ligand pathways could provide additional mechanistic insight into the role of trained immune processes in SSc pathogenesis.

In our cohort, unsupervised clustering of significantly expressed biomarkers classified patients into a low- and a high-inflammatory endotype (Figure 2). Interestingly, patients within the high-inflammatory endotype were older and had longer disease durations, while esophagitis was less prevalent (Figure 2a). Correlation analysis further supported these results, as several proteins (TNF, CXCL9, TNFRSF9, and CXCL10) were associated with disease progression (Figure 3.). From one perspective, the higher protein concentrations could merely reflect aging. The aging process has been associated with chronic low-grade inflammation and the enhanced production of various cytokines, including TNF, IL-6, IL-1, and IL-8 [34,35]. Furthermore, the recent work by Sayed et al. labeled CXCL9 as a marker of age-related chronic inflammation that reflects cellular senescence and vascular dysfunction [36]. Consistent with previous data, in addition to CXCL9, CXCL10 followed a similar trend, demonstrating higher levels in elderly patients with Chagas disease [37]. Similarly, in a murine model, single-cell RNA sequencing and multicolor flow cytometry identified age-related dynamic changes in the composition of the CD4 T cell compartment. A decline in naive T cells and gradual accumulation of cytotoxic, exhausted, and activated regulatory T cells (T_regs_) appeared with age. Activated T_regs_ express increased levels of the costimulatory gene TNFRSF9 and its protein [38]. Conversely, these results could highlight a persistent Th 1 signature as the disease progresses in contrast with former published data. Hence, in systemic sclerosis, a skewed balance between Th 1 and Th 2 cytokines with a shift from an early inflammatory (Th 1) immune response to a later profibrotic (Th 2) immune phenotype has been supported by several lines of evidence [39,40,41]. Remarkably, in a cohort of 37 patients with SSc, CXCL10 (Th 1) serum levels declined during follow-up, while CCL2 (Th 2) remained stable [18]. Furthermore, in a longitudinal study of 26 patients with early diffuse cutaneous SSc, a decline in serum concentrations of Th 2 cytokines along with an increase in IL-12, a Th 1 cytokine, was associated with regression in skin sclerosis [42]. Collectively, the circulating proteins in our study population could either reflect protracted inflammatory activity despite disease progression or rather depict a cohort of patients with fewer fibrotic manifestations and a better SSc prognosis.

SSc remains a clinical diagnosis that is often difficult to establish, especially in the early phases when non-specific vascular manifestations, such as Raynaud’s phenomenon, dominate the clinical picture followed only later by skin sclerosis and internal organ involvement. The identification of relevant biomarkers that could aid in a timely diagnosis or more accurately predict disease complications is therefore justified. Our study provides additional evidence for the utility of circulating proteins in SSc diagnosis. The best predictive accuracy was obtained for the multi-protein panel encompassing TNF, CXCL9, CXCL10, and CX3CL1, even though satisfactory diagnostic performance was registered for the individual proteins as well (Figure 4). Given our limited sample size, we were not able to determine the discriminative performance of the identified analytes in classifying internal organ manifestations. However, Bauer et al. validated a panel of eight proteins derived from the serum proteome (collagen IV, endostatin, insulin-like growth factor binding protein (IGFBP)-2, IGFBP-7, matrix metallopeptidase-2, neuropilin-1, N-terminal pro-brain natriuretic peptide, and receptor for advanced glycation end products) that distinguished SSc patients with PAH (*n* = 77) from SSc patients without PAH (*n* = 80) in two independent cohorts (AUC = 0.741, sensitivity = 65.1%, specificity = 69.0% in the DETECT Discovery Cohort and AUC = 0.811, sensitivity = 77.3%, specificity = 86.5% in the Sheffield Confirmatory Cohort) [43].

There have been few other studies employing the Olink platforms for proteomic analyses in patients with SSc. Accordingly, Farutin et al. [6] explored a large number of proteins using 11 panels encompassing 981 biomarkers and identified 70 circulating proteins involved in both fibrotic and inflammatory processes. Further analysis showed that 39 analytes were associated with more severe skin disease, including the upregulation of some extracellular matrix components (NOV, THBS4) and downregulation of several growth factor receptors (epidermal growth factor receptor, Delta/notch-like epidermal growth factor-related receptor) and adhesion molecules (integrin subunit alpha V) [6]. More recently, Castro et al. [44] assessed the levels of serum proteins from a panel of 92 analytes related to organ damage in a cohort of 72 patients with SSc. Sixteen differentially expressed biomarkers successfully classified patients into three clusters. Cluster one was distinguished from clusters two and three based on the more prevalent internal organ involvement, mainly ILD, skin thickening, and esophageal dysmotility, as well as a higher frequency of anti-Scl-70 antibodies. Also, after performing unsupervised hierarchical analysis, nineteen new circulating proteins (BANK1, BID, CALR, ERBB2IP, FGR, FOSB, FOXO1, INPPL1, MAEA, MAGED1, MAP4K5, NBN, NCF2, PRKAB1, RASSF2, RCOR1, SMAD1, STXBP3, VASH1) involved in various molecular pathways were found to be differentially expressed between the clusters [44].

The present study has several limitations. Firstly, the small sample size does not reflect the heterogeneity of the SSc population, and together with missing data, this hindered our ability to establish relevant clinical associations. Secondly, serum protein assessment was restricted to the analytes included in the Olink^®^ Target 96 Inflammation Panels, and therefore, expanding the analysis to larger numbers of candidate biomarkers could potentially unravel novel molecular pathways and disease endotypes. Moreover, using a stringent statistical approach limited the risk of false discoveries and enabled us to identify reliable analytes but might have also resulted in missing relevant circulating proteins that did not reach statistical significance. Thirdly, the lack of external validation impairs the reproducibility and generalizability of the obtained results. Lastly, the cross-sectional design of our work does not reflect changes in protein expression levels over time, thus highlighting the need for larger studies with longitudinal data.

## 4. Materials and Methods

### 4.1. Study Design, Setting, and Participants

Thirty-six participants (18 SSc patients and 18 age- and gender-matched controls) were included between October 2021 and March 2022 in this cross-sectional case-control study. Ethical approval was granted by the Ethics Committee of “Iuliu Hațieganu” University of Medicine and Pharmacy, Cluj-Napoca, under the following reference number: 104/09.03.2020. Patients attending the Rheumatology Department at County Emergency Hospital Cluj-Napoca had to fulfill the 2013 American College of Rheumatology/European League against Rheumatism SSc Classification Criteria to be enrolled, while healthy controls without a previous history of systemic connective tissue disease or inflammatory arthritis were recruited from the hospital personnel. All participants provided written informed consent, and the study was conducted in accordance with the Declaration of Helsinki.

### 4.2. Variables and Data Collection

Baseline patient demographics and clinical characteristics were retrieved from medical records. The mRSS was used to evaluate the degree of skin involvement and further categorize patients into limited cutaneous or diffuse cutaneous SSc subsets based on the criteria established by LeRoy et al. [45]. The presence of ground-glass opacifications, reticular changes, traction bronchiectasis, or honeycombing in a bibasilar distribution on HRCT scans was considered diagnostic for ILD. Screening for PAH and referral for right heart catheterization (RHC) adhered to the guidelines outlined by the 2015 European Society of Cardiology/European Respiratory Society [46]. Disease duration, measured from the onset of the first non-Raynaud symptom to blood sample collection, was categorized as either early (≤3 years) or late (>3 years).

### 4.3. Sample Collection and Assessment of Inflammation Biomarkers

Venous blood was collected from a cubital vein into ethylenediaminetetraacetic acid (EDTA) tubes under sterile conditions. After centrifugation for 10 min at 1700× rpm, plasma samples were stored at −80°C and shipped on dry ice to Radboud University Medical Center, Nijmegen, The Netherlands for proteome analysis using the Olink^®^ Target 96 Inflammation Panels (Olink Bioscience, Uppsala, Sweden). Proximity Extension Assay (PEA) technology was employed for the detection of the 92 plasma proteins included in the Inflammation panel, following the manufacturer’s instructions. Protein concentrations were expressed as NPX, Olink^®^’s relative quantification unit, on a log2 scale. A difference of 1 NPX translates into a doubling of the protein concentration, and a high NPX value corresponds to a high biomarker concentration. Pre-processing of the data was achieved using the reference sample normalization method. Protein expression data were IPC-normalized to adjust for non-specific binding and background noise. Built-in internal and external controls were used for data quality checks with samples flagged as “Warning” by the Olink^®^ QC system being excluded from further analysis. Proteins with expression levels below the LOD in more than 50% of the samples were removed from subsequent data processing.

### 4.4. Statistical Analysis

Baseline demographics and clinical characteristics were outlined employing descriptive statistics. The median and IQR were used to report continuous variables, while categorical variables were expressed as frequencies and percentages. Comparisons between groups were performed using the Wilcoxon rank-sum test for ordinal data or the Chi-square test for binary data. Following data distribution, Spearman’s rank or Pearson’s correlation coefficients were applied to measure the strength of the association between two variables. The R package “Olink^®^ Analyze” was employed for differential expression analysis of plasma proteins between the two groups (patients with SSc versus healthy controls) using Welch’s two-sample, two-sided *t*-test. The FDR was reduced by applying the Benjamini–Hochberg correction of multiple testing with proteins reaching the level of significance if the FDR-adjusted *p*-value was <0.05. Volcano plots were generated to visualize the differentially expressed proteins. PCA analysis was used to dimensionally reduce the data and, together with the heatmaps, to visualize data patterns and relationships between the samples or proteins. All statistical analysis was performed with R software (4.2.3 version) with R Studio IDE (as an integrated development environment). Base packages were as follows: Olink^®^ Analyze (3.4.1), xlsx (0.6.5), stats (base package), dplyr (1.1.2), ggplot2 (3.4.2), cowplot (1.1.1), running under Windows 10 × 64, 22H2 (build19045).

## 5. Conclusions

In summary, our targeted proteomic analysis revealed an individual protein signature comprising mainly chemokines, potent molecular mediators of angiogenesis that promote vascular dysfunction and remodeling in SSc, together with members of the TNF superfamily (TNFSF) and TNF receptor superfamily (TNFRSF). The enhanced expression of cytokines and costimulatory receptors of TNFSF and TNFRSF observed in our cohort, as well as their involvement in the activation and metabolic rewiring of innate immune cells documented in the literature, supports a role for a perpetual proinflammatory milieu contributing to the etiopathogenesis of SSc. Aging and longer disease courses were associated with prominent inflammatory activity, while polarization towards a Th1 immune response proved to persist irrespective of the disease duration. Lastly, circulating proteins exhibited high diagnostic accuracy, performing therefore as promising diagnostic biomarkers. Even though these data require further validation in larger cohorts, the identified proteins have important functions in innate immune responses and vasculopathy that may be of great relevance to SSc pathogenesis.

## Figures and Tables

**Table 1:** Baseline demographics of patients with SSc.

Characteristics	SSc, n = 15 ^1^
**Age**	53 (48, 60)
**Gender**	
female	15 (100%)
**SSc subtype**	
diffuse cutaneous	6 (40%)
limited cutaneous	9 (60%)
**Disease duration** (years)	4 (0.5, 9.0)
**Disease duration** (early ≤ 3 years; late >3 years)	
early	7 (47%)
late	8 (53%)
**Calcinosis**	2 (13%)
**Telangiectasis**	9 (60%)
**Digital ulcers** (previous, current, never)	
previous	7 (47%)
current	2 (13%)
never	6 (40%)
**ILD**	8 (53%)
**Arrythmias requiring therapy**	4 (27%)
**Conduction blocks**	2 (13%)
**PAH requiring therapy**	1 (6.7%)
**Esophagitis**	5 (33%)
**ANA**	
positive	15 (100%)
**anti-Scl-70 antibodies**	
positive	8 (53%)
**anti-centromere antibodies**	
positive	5 (33%)

^1^ n (%); median (IQR). SSc = systemic sclerosis; ILD = interstitial lung disease; PAH = pulmonary arterial hypertension; ANA = antinuclear antibodies.

**Table 2:** Pearson’s correlation coefficient and level of significance of the differentially abundant proteins.

Variable	Pearson’s Coefficient	*p*-Value
TNF	0.6615	0.0072
CXCL9	0.6371	0.0106
TNFRSF9	0.5697	0.0266
CXCL10	0.5365	0.0392
LIF-R	0.4778	0.0716
CXCL11	0.4188	0.1203
CD40	0.3497	0.2013
HGF	0.3129	0.2562
CCL28	0.2872	0.2993
TNSF14	0.2814	0.3097
CCL19	0.2770	0.3175
CCL4	0.2463	0.3763
MCP-4	0.2196	0.4315
CXCL6	0.1976	0.4801
CCL11	0.1439	0.6090
CX3CL1	0.0891	0.7523
CCL23	0.0008	0.9978

## Data Availability

Data are contained within the article.
